# Cowpea viruses: Effect of single and mixed infections on symptomatology and virus concentration

**DOI:** 10.1186/1743-422X-4-95

**Published:** 2007-09-27

**Authors:** Moni A Taiwo, Kehinde T Kareem, Imade Y Nsa, Jackies D'A Hughes

**Affiliations:** 1 Dept. of Botany and Microbiology, University of Lagos, Akoka, Lagos, Nigeria; 2 International Institute of Tropical Agriculture, Ibadan, Nigeria

## Abstract

Natural multiple viral infections of cultivated cowpeas have been reported in Nigeria. In this study, three Nigerian commercial cowpea cultivars ("Olo 11", "Oloyin" and "White") and two lines from the IITA (IT86D- 719 and TVU 76) were mechanically inoculated with *Cowpea aphid-borne mosaic virus *(CABMV), *Bean southern mosaic virus *(SBMV) and *Cowpea mottle virus *(CMeV) singly, as well as in all possible combinations at 10, 20 and 30 days after planting (DAP). Samples of leaves or stems were collected at 10, 20 and 30 days after inoculation (DAI) and analyzed for relative virus concentration by Enzyme-Linked Immunosrbent Assay. All the cultivars and lines {CVS/L} were susceptible to the viruses but the commercial CVS showed more severe symptoms and had relatively higher viral concentration. In single virus infections, CABMV which induced the most severe symptoms had absorbance values (at 405 nm) of 0.11 to 0.46 while SBMV and CMeV which induced moderate symptoms had virus titre of 0.74 to 1.99 and 0.11 to 0.90 respectively. Plants inoculated 10 DAP had significantly higher virus concentration than those inoculated 30 DAP. In mixed infections involving CABMV (10 DAP) apical necrosis and death were observed in commercial cultivars "Olo 11" and "White". Enhancement of CMeV titers were observed in plants infected with CMeV + CABMV. Multiple viral infections of cowpeas may result in complete yield loss, hence, the availability of seeds of cultivars with a high level of multiple virus resistance is recommended as a means of control.

## 1.0 Background

Estimated yield losses due to viral infection of cowpeas are between 10% and 100% [1]. Presently, the use of resistant varieties is the most economical, practicable and effective method of controlling the viruses [2]. Cowpea lines with individual and combined resistance to several cowpea viruses have been identified at the International Institute of Tropical Agriculture (IITA) and tested for local adaptation [3]. In spite of this, viruses are still detected on commercially cultivated cowpeas in Nigeria [4]. In a recent survey, Shoyinka *et al*., [5] reported that there was no ecological restriction to the distribution of the six viruses detected. *Cowpea aphid-borne mosaic virus *(CABMV) genus *Potyvirus *and *Bean southern mosaic virus *(SBMV) genus *Sobemovirus *were highly prevalent but had moderate incidence while *Cowpea mottle virus *(CMeV) genus *Carmovirus *was moderate in both incidence and prevalence.

Natural multiple infections caused by 4–5 viruses were also observed but those caused by two viruses were most prevalent [5]. Mixed viral infections have biological, epidemiological and economic implications [6,7]. Viruses in mixed infections may interact synergistically or antagonistically [8-11] causing changes in the concentration of either or both viruses [12,13] and consequently causing a new disease [14]. Apart from the synergistic interaction between CABMV and *Cucumber mosaic virus *(CMV) genus *Cucu movirus *reported by Pio- Ribeiro *et al*., [14] and the quantitative and qualitative effects of single and mixed viral infections on cowpeas [15,16], very limited information is available on the interactive effects of mixed viral infections on cowpeas.

This study was initiated to document the symptoms induced in three Nigerian commercial cowpea cultivars and two breeding lines from IITA as a result of single and mixed inoculations with three cowpea viruses (CABMV, CMeV, SBMV), establish if symptomatology was correlated with relative virus concentration and ascertain if there are any interactions between the viruses [17].

## 2.0 Materials and methods

### 2.1. Sources of viruses and cowpea cultivars/lines (CVS/L)

One isolate each of CABMV, CMeV and SBMV and the two cowpea lines (IT86D-719 and TVU 76) used for this investigation were obtained from IITA. The virus isolates which were previously stored over CaCl2 at 4°C were propagated and subsequently maintained on cowpea cultivar "Ife Brown". Seeds of the commercial cowpea cultivars ("Oloyin", "Olo 11" and "White") were obtained from and confirmed as released varieties at the Federal Ministry of Agriculture, Moor Plantation, Ibadan. Seeds of the different CVS/L were planted in labeled plastic pots and maintained in a greenhouse at 28–35°C, at the University of Lagos.

### 2.2. Virus treatments

Mechanical inoculations were performed 10 days after planting (DAP) with the following inocula: CABMV, CMeV, SBMV, CABMV+CMeV, CABMV+SBMV, CMeV+SBMV, CABMV+CMeV+SBMV and buffer (control). The treatments were repeated with other sets of plants inoculated 20 and 30 DAP between October and November 2002.

Viral inocula were prepared by grinding systemically infected leaves from cowpea cultivar "Ife Brown" infected with individual viruses (1:2 w/v) in a sterilized mortar with pestle in 0.05 M K2HPO4 pH 7.5. For mixed viral treatments, saps from the relevant inocula were mixed in ratio 1:1 (V/V) just before inoculation. The plants were dusted with Carborundum before inoculation. After inoculation, the pots were arranged in a randomized complete block design with three (3) replications. There were three blocks, each block consisted of 120 plastic pots and represented the plants inoculated on 10, 20 and 30 DAP. The pots were kept in a greenhouse that was sprayed weekly with cypermetrin 10% E.C, and were observed for symptoms at 10 days interval until flowering.

### 2.3. Virus titer determination

Young leaf samples of about the same age were plucked from the same position and at times stems of dying plants that received the various treatments at 10, 20 and 30 days after inoculation (DAI). The samples were kept in grinding pouches (Agdia Inc. Elk. IN, USA) and stored in the freezer (-4°C), until the end of the experiment. The samples from the various treatments were weighed on a weighing balance (Mettler Toledo, Switzerland), ground in extraction buffer (0.05 M sodium carbonate buffer (pH 9.6) with 2 % (wt/vol) Polyvinylpyrrolidone) and analyzed by antigen-coated plate enzyme-linked immunosorbent assay (ACP-ELISA) at IITA according to Koenig [18]. Samples were considered positive when the absorbance value (at 405 nm) were at least twice that of the mean for the negative control. The average of the absorbance values (at 405 nm) from the samples taken from plants that received similar treatments was determined and recorded.

### 2.4 Statistical analysis

The statistical package for social scientists (SPSS) was used for the analysis of the data obtained. Tukey HSD test was used to determine the level of significance between the cultivars/lines and virus treatments.

## 3.0 Results

### 3.1. Response of Cowpea CVS/L to viral treatments

All the commercial cowpea cultivars and IITA lines used in this investigation were susceptible to the three viruses. Systemic symptoms which varied from green-vein banding to mosaic, mottle, internode shortening, apical necrosis and reduction in leaf size were induced in plants that were inoculated singly with CABMV, CMeV or SBMV, depending on the age of the plant at the time of inoculation (Table 1 - for tables, see Additional file 1). Some of the cultivars ("Olo 11", "White", TVU 76) that were inoculated with a mixture of two viruses (CABMV+SBMV or CABMV+CMeV) at 10 DAP died prematurely while the other CV/L were stunted with completely reduced leaf size (Table 1). Plants inoculated with a mixture of the three viruses 10 DAP also showed severe symptoms resulting in apical necrosis and reduction in leaf size. On the basis of the cultivars' response to the viruses in single and mixed infections, "Olo 11", "White" and TVU 76 appeared to be more susceptible than "Oloyin".

**Figure 1:**
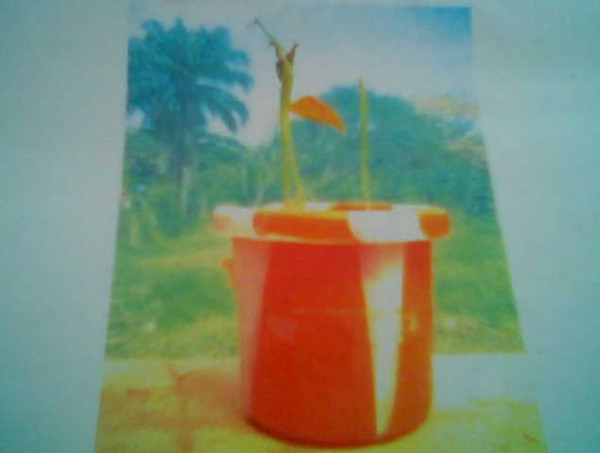
Apical necrosis induced on plants inoculated with a combination of the three viruses.

CABMV was the most aggressive of the three viruses. It induced the most severe symptoms especially in mixed infections with CMeV or SBMV at an early stage of growth (10 DAP). Most of the plants inoculated at this stage died prematurely. The plants inoculated at the later stages (20 and 30 DAP) showed mild symptoms only, however, those that were inoculated with a combination of the three viruses developed apical necrosis (Fig. 1).

### 3.2. Virus concentration in plants infected by single viruses

The age of plant at the time of infection as well as the CVS/L had significant effect on the titer of CABMV in the infected plants. The concentrations of CABMV in IT86D-719 when singly infected at 10 and 30 DAP were significantly higher than those of TVU 76 while there were no significant differences between the titres of "White" and "Oloyin". Absorbance values (at 405 nm) ranging from 0.11 to 0.46 were observed for CABMV. The titer of CMeV in single virus infection with "oloyin" was significantly low compared to other CVS/L. However, the concentrations in "Olo 11" were not significantly different from those of IT86D- 719 and "White" at 10 and 30 DAP respectively. The absorbance values (at 405 nm) of CMeV for all CVS/L ranged from 0.11 to 0.90. The titer of SBMV was very high in all the CVS/L tested with concentrations ranging from 0.74 to 1.99. Moreso, the titres of SBMV in IITA lines were significantly lower than those of commercial cultivars (Table 2). Generally, for all the viruses and CVS/L, absorbance values from plants inoculated 10 DAP were significantly higher than those from plants inoculated 30 DAP (Table 2).

### 3.3 Virus titer in mixed infections

In mixed virus infections involving CABMV and CMeV or SBMV, the concentration of the Potyvirus component (CABMV) remained virtually unchanged in the different cowpea CVS/L. Statistically, the concentrations of CABMV in both single and dual infections were not significantly different (Tables 3 and 4). In CABMV+CMeV infections, the ratios of dual/single (CABMV) infections ranged from 0.94 to 1.18 with IT86D-719 having the highest titre while TVU 76 had the least (Table 3). The concentration of CMeV in dual infection was significantly higher than in single infection. Similarly, for CABMV+SBMV infections, the ratios of dual/single (CABMV) infections ranged from 1.00 to 1.11 (Table 4). However, an enhancement in the titer of CMeV in CMeV+CABMV infection was observed, with the ratio of dual/single infections ranging from 1.11 to 3. The enhancement of CMeV titer was most evident in "Oloyin" (Table 3). There was no evidence of enhancement of the titer of SBMV in SBMV+CABMV infection, as the ratio of SBMV in dual/single infection varied from 0.7 to 1.54 only (Table 4). In triple virus infections, the three viruses were detected in all the cowpea CVS/L. Some of the plants inoculated 10 DAP died prematurely (Table 1).

## 4.0 Discussion

The results of this study have shown that the three Nigerian commercial cultivars (Olo 11, White, Oloyin) used in this investigation are susceptible to CABMV, CMeV and SBMV. Owolabi *et al*., [15] had previously reported the susceptibility of two other Nigerian commercial cowpea cultivars to *Cowpea mosaic virus *genus *Comovirus *and *Blackeye cowpea mosaic virus *genus *Potyvirus*. In this study, the commercial cultivars did not only show a more severe response to the various viral treatments, they also appeared to have a relatively higher virus concentration than the IITA breeding lines. This suggests that where immunity to a cowpea virus cannot be identified, resistance breeding may be enhanced by the determination of virus titer in the screened plant.

Generally, viral infection of cowpea at an early age resulted in more severe symptoms, sometimes resulting in death of the affected plants. This is corroborated in this investigation, by the higher concentration of the viruses in plants infected 10 DAP. Such an early infection of cowpeas during the hot and dry conditions associated with the dry season may result in complete loss of yield [19]. A similar observation was reported in field grown cowpeas in Northern Nigeria by Raheja and Leleji [20]. Also, studies by Taiwo and Akinjogunla [16] have confirmed that infection of cowpeas at such an early age of 10 DAP resulted in a greater reduction in the growth and yield parameters as well as the nutritive content of the seeds, compared with those of plants infected at maturity.

In single virus infections, CABMV induced the most severe symptom of the three viruses but its concentration was least in most cases. In mixed virus infections involving CMeV and CABMV, the titer of CMeV was always higher than its corresponding titer in single infections. This suggested some form of synergistic interaction between CABMV and CMeV. The enhancement in CMeV titer was detected in all the cultivars although it was more pronounced in two of the commercial CVS ("Oloyin" and "White"). The synergism observed is further confirmed by the increased symptoms observed in CVS "Olo 11" and "White" inoculated with a mixture of CABMV and CMeV. *Potyvirus *synergism has been reported by a number of workers [21,12,22,11]. Anjos *et al*., [9], showed that *Soybean mosaic virus *(SMV) genus *Potyvirus *interacted synergistically with some comoviruses, but two other potyviruses, *Bean yellow mosaic virus *and *Peanut mottle virus *did not, suggesting that not all potyviruses are involved in the synergistic interaction.

In these interactions, the concentration of the *Potyvirus *member remained unchanged while the concentration of the non-*Potyvirus *member increased significantly, in the dually infected plants [9,11]. A number of mechanisms have been proposed for the synergism between comoviruses and potyviruses. These include the ability of the comoviruses to utilize the replication machinery of the *Potyvirus *(SMV) for their multiplication, since the two groups have been shown to share some amino acid sequences [23,24]. Also, the SMV enclosed movement protein has been implicated in enhancing the transportation of the *Comovirus *and by so doing increasing the number of infected cells in dually infected plants [9].

Although CMeV belongs to the genus *Carmovirus*, it has isometric particles like the comoviruses. The mechanism for the enhancement of its titer needs to be determined as there are no previous reports of such interactions, or similarity in genomes of potyviruses and carmoviruses. Interestingly, the titer of SBMV, another isometric virus was not enhanced by CABMV during this investigation.

These results confirm the susceptibility of Nigeria's commercial cowpea cultivars to viral infections, in spite of several reports on the availability of sources of resistance to the viruses [3,4,2]. Early infection of the cultivars by multiple viruses especially with CABMV may result in complete loss in yield. The implication of this result is that either the rate of acceptance and utilization of resistant varieties in Nigeria is poor or new resistance breaking strains of the viruses have evolved. There is a need to intensify efforts at continuously monitoring the predominant field virus strains, and developing advanced cowpea breeding lines/CVS with multiple resistance to the economically important viruses. The seeds of the resistant cultivars should possess horticultural and culinary desirable traits, and should be readily available to growers, in order to minimize losses due to viral infections. There may also be the need to explore other control strategies such as pathogen-derived resistance in the management of cowpea viruses. The modern concept of production of transgenic plants that has been applied to tobacco and papaya [25,26] may have to be adapted to cowpea, for effective virus control and sustenance of the nation's lead in cowpea production.

## Supplementary Material

Additional File 1

## References

[B1] Rachie KO, Singh SR, Rachie KO (1985). Introduction. Cowpea research, production and utilization.

[B2] Taiwo MA, Hughes J d'A, Odu, B (2003). Viruses infecting legumes in Nigeria: case history. Plant Virology in Sub-Saharan Africa.

[B3] Thottappilly G, Rossel HW (1992). Virus diseases of cowpea in tropical Africa. Tropical Pest Management.

[B4] Huguenots C, Furneaux MT, Thottappilly G, Rossel HW, Hamilton RI (1993). Evidence that *Cowpea aphid- borne mosaic *and *Blackeye cowpea mosaic viruses *are two different potyviruses. J Gen Virol.

[B5] Shoyinka SA, Thottappilly G, Adebayo GG, Anno-Nyako FO (1997). Survey on cowpea virus incidence and distribution in Nigeria. International J of pest management.

[B6] Rochow WF (1972). The role of mixed infections in the transmission of plant viruses by aphids. Annu Rev Phytopathol.

[B7] Ford RE, Goodman RM, Hill LD (1976). Epidemiology of soybean viruses. World Soybean Res Conf 1975.

[B8] Kassanis B (1963). Interaction of viruses in plants. Adv Virus Res.

[B9] Anjos JR, Jarlfors U, Ghabrial SA (1992). Soybean mosaic Potyvirus enhances the titer of two Comoviruses in dually infected soybean plants. Phytopathology.

[B10] Vance VB, Berger PH, Carrington JC, Hunt AG, Shi XM (1995). 5 proximal potyviral sequences mediate potato X potyviral synergistic disease in transgenic tobacco. Virology.

[B11] Murphy JF, Bowen KL (2006). Synergistic disease in pepper caused by the mixed infection of *Cucumber mosaic virus *and *Pepper mottle virus*. Phytopathology.

[B12] Calvert LA, Ghabrial SA (1983). Enhancement by *Soybean mosaic virus *of *Bean pod mottle virus *titer in doubly infected soybean. Phytopathology.

[B13] Goldberg K, Brakke KM (1987). Concentration of *Maize chlorotic mottle virus *increased in mixed infections with *maize dwarf mosaic virus*, strain B. Phytopathology.

[B14] Pio-Ribeiro G, Wyatt SD, Kuhn CW (1978). Cowpea stunt: a disease caused by the synergistic interaction of two viruses. Phytopathology.

[B15] Owolabi AT, Taiwo MA, Mabadeje SA (1988). Effects of single and mixed inoculations with *Blackeye cowpea mosaic *and *Cowpea mosaic viruses *on two Nigerian cowpea cultivars. Nigerian J of Basic and Applied Sci.

[B16] Taiwo MA, Akinjogunla OJ (2006). Cowpea viruses: Quantitative and Qualitative effects of single and mixed viral infections. African J of Biotech.

[B17] Taiwo MA, Apampa K, Hughes d'AJ, Nsa IY (2006). Cowpea viruses: Effect of single and mixed infections on symptomatology and virus concentration. Phytopathology.

[B18] Koenig R (1981). Indirect ELISA methods for the broad specificity detection of plant viruses. J Gen Virol.

[B19] Kareem KT, Taiwo MA (2007). Interactions of viruses in cowpea: effects on growth and yield parameters. Virology J.

[B20] Raheja AK, Leleji OI (1974). An aphid-borne virus disease of irrigated cowpea in northern Nigeria. Plant Dis Rep.

[B21] Ross JP (1968). Effect of single and double infections of *Soybean mosaic *and *Bean pod mottle viruses *on soybean yield and seed character. Plant Dis Rep.

[B22] Wang Y, Gaba V, Yang J, Palukaitis P, Gal-on A (2002). Characterization of synergy between *Cucumber mosaic virus *and potyviruses in cucurbit hosts. Phytopathology.

[B23] Domier L, Shaw JG, Rhoads RE (1987). Potyviral proteins share amino acids homology with picorna-, como-, and caulimoviral proteins. Virology.

[B24] Goldbach R (1987). Genome similarities between plant and animal RNA viruses. Microbiol Sci.

[B25] Powell-Abel P, Nelson RS, De B, Hoffmann N, Roggers SG, Fraley RT, Beachy RN (1986). Delay of disease development in transgenic plants that express the *Tobacco mosaic virus *coat protein gene. Science.

[B26] Bau HJ, Cheng YH, Yang JS, Yeh SD (2003). Broad spectrum resistance to different geographic strains of *Papaya ringspot virus *in coat protein transgenic Papaya. Phytopathology.

