# Supplementary material for: Cowpea viruses: Effect of single and mixed infections on symptomatology and virus concentration
Source: Virol J. 2007 Sep 27;4:95. doi: 10.1186/1743-422X-4-95 (PMC2222634; doi:10.1186/1743-422X-4-95)
Supplement: Additional File 1 [file 1743-422X-4-95-S1.doc]

Table 1: Symptoms induced in 5 cowpea cultivars/lines inoculated with cowpea viruses singly and in mixtures.

| **TREATMENTS** | **COWPEA CULTIVARS/LINES** | | | | |
| --- | --- | --- | --- | --- | --- |
|  | **OLOYIN** | **OLO 11** | **WHITE** | **TVU 76** | **IT86D-719** |
| CABMV* | **GVB,RL | GVB,AN | GVB,DE | GVB,IS | GVB,IS |
| CMeV | Mo,IS | Mo,IS | Mo,Df | Mo | Mo, RL |
| SBMV | B,M | B,LC | B,M,Df | M,LC | M, LC |
| CABMV+CMeV | Mo,AN | Mo,DE | AN,DE | Mo,AN | Mo,LC |
| CABMV+ SBMV | M,B | GVB, DE | M, B | M.RL | AN, IS |
| CMeV +SBMV | Mo,M | Mo,M | RL,Df | Mo,D | Mo,LC,Df |
| CABMV+CMeV+SBMV | Mo,M,AN | AN,IS | AN,D | AN,D | AN,D |

*CABMV = *Cowpea aphid-borne mosaic virus*, CMeV = *Cowpea mottle virus*,

SBMV = *Bean Southern mosaic virus.*

** GVB = Green-vein banding, RL = Reduction in leaf size, AN = Apical necrosis,

DE = Death of plants inoculated at an early age, IS = Internodes shortening, Mo = Mottle, Df = defoliation, B = Blistering, M = Mosaic, LC = Leaf curl, D = Death,

Table 2: Virus concentration determined by enzyme-linked immunosorbent assay

(ELISA) for cowpea plants inoculated with three viruses individually.

| **ELISA values (at 405nm)** | | | | | | |
| --- | --- | --- | --- | --- | --- | --- |
| Virus inocula** | | | | | | |
| Cowpea | CABMV | | CMeV | | SBMV | |
| CVS/L***** | 10 | 30 | 10 | 30 | 10 | 30 |
| White | 0.280^c^ | 0.158 ^bc^ | 0.901^a^ | 0.300^a^ | 1.992^a^ | 0.947^c^ |
| Olo 11 | 0.464^a^ | 0.220^b^ | 0.450^b^ | 0.303^a^ | 1.488^b^ | 1.183^a^ |
| Oloyin | 0.274^c^ | 0.163^bc^ | 0.134^d^ | 0.110^c^ | 1.416^c^ | 1.105^b^ |
| TVU 76 | 0.249^cd^ | 0.110^c^ | 0.262^c^ | 0.244^ab^ | 1.344^d^ | 0.813^d^ |
| IT86D-719 | 0.384^b^ | 0.344^a^ | 0.425^b^ | 0.192^b^ | 1.341^d^ | 0.741^c^ |
| Control | 0.080^d^ | 0.080^d^ | 0.018^c^ | 0.095^d^ | 0.119^c^ | 0.119^f^ |

Letters (superscript) following means within a column, when different, indicate a significant difference according to Tukey HSD test (P<0.05).

Mean ELISA absorbance value at 405nm for leaf samples inoculated at 10 and 30 days after planting (DAP).

****** CABMV = *Cowpea aphid-borne mosaic virus.* CMeV = *Cowpea mottle virus.* SBMV = *Bean southern mosaic virus*.

* Cultivars/Lines

Table 3: Comparison of titers of *Cowpea aphid-borne mosaic virus* (CABMV) and *Cowpea mottle virus* (CMeV) in single and dual infections, as determined by enzyme-linked immunosorbent assay (ELISA).

| ELISA values (at 405nm)^#^ | | | | | | |
| --- | --- | --- | --- | --- | --- | --- |
| Cowpea  CVS/L* | CABMV Alone + CMeV | | Ratio****** | CMeV Alone + CABMV | | Ratio******* |
| White | 0.175 ^c^ | 0.190 ^c^ | 1.08 | 0.518^b^ | 1.154^a^ | 2.23 |
| Olo 11 | 0.225 ^c^ | 0.243 ^c^ | 1.08 | 0.334^b^ | 0.507^a^ | 1.52 |
| Oloyin | 0.241^b^ | 0.226^b^ | 0.94 | 0.130 ^c^ | 0.391^a^ | 3.00 |
| TVU 76 | 0.131 ^c^ | 0.134^C^ | 1.02 | 0.262^b^ | 0.406^a^ | 1.55 |
| IT86D-719 | 0.365^a^ | 0.434^a^ | 1.18 | 0.319^b^ | 0.354^a^ | 1.11 |

Letters (superscript) following means across a row, when different, indicate a significant difference according to Tukey HSD test (P<0.05).

^#^ Mean ELISA absorbance value at 405nm for leaf samples inoculated at 20 days after planting (DAP).

CABMV = *Cowpea aphid-borne mosaic virus*. CMeV = *Cowpea mottle virus*.

*Cultivars/Lines

**Ratio of absorbance values (at 405nm) of CABMV in CABMV+CMeV/CABMV infections

***Ratio of absorbance values (at 405nm) of CMeV in CMeV+CABMV/CMeV infections

Table 4: Comparison of titers of *Cowpea aphid-borne mosaic virus* (CABMV) and *Bean southern mosaic* *virus* (SBMV) in single and dual infections, as determined by enzyme-linked immunosorbent assay (ELISA).

| ELISA values (at 405nm)^#^ | | | | | | |
| --- | --- | --- | --- | --- | --- | --- |
| Cowpea  CVS/L* | CABMV Alone + SBMV | | Ratio****** | SBMV Alone + CABMV | | Ratio******* |
| White | 0.175^c^ | 0.180 ^c^ | 1.03 | 1.201^a^ | 0.845^b^ | 0.70 |
| Olo 11 | 0.225 ^c^ | 0.225 ^c^ | 1.00 | 1.024^b^ | 1.207^a^ | 1.18 |
| Oloyin | 0.241 ^c^ | 0.243 ^c^ | 1.01 | 1.105^b^ | 1.328^a^ | 1.20 |
| TVU 76 | 0.131 ^c^ | 0.124 ^c^ | 1.06 | 0.813^a^ | 0.620^b^ | 0.76 |
| IT86D-719 | 0.365 ^c^ | 0.405 ^c^ | 1.11 | 0.836^b^ | 1.284^a^ | 1.53 |

Letters (superscript) following means across a row, when different, indicate a significant difference according to Tukey HSD test (P<0.05).

^#^ Mean ELISA absorbance value at 405nm for leaf samples inoculated at 20 days after planting (DAP).

CABMV = *Cowpea aphid-borne mosaic virus*. SBMV = *Bean southern mosaic virus.*

*Cultivars/Lines

**Ratio of absorbance values (at 405nm) of CABMV in CABMV+CMeV/CABMV infections

***Ratio of absorbance values (at 405nm) of SBMV in SBMV+CABMV/SBMV infections.
